# FoxM1 Is a General Target for Proteasome Inhibitors

**DOI:** 10.1371/journal.pone.0006593

**Published:** 2009-08-12

**Authors:** Uppoor G. Bhat, Marianna Halasi, Andrei L. Gartel

**Affiliations:** 1 Department of Medicine, University of Illinois at Chicago, Chicago, Illinois, United States of America; 2 Department of Biochemistry and Molecular Genetics, University of Illinois at Chicago, Chicago, Illinois, United States of America; 3 Department of Microbiology and Immunology, University of Illinois at Chicago, Chicago, Illinois, United States of America; Dresden University of Technology, Germany

## Abstract

Proteasome inhibitors are currently in the clinic or in clinical trials, but the mechanism of their anticancer activity is not completely understood. The oncogenic transcription factor FoxM1 is one of the most overexpressed genes in human tumors, while its expression is usually halted in normal non-proliferating cells. Previously, we established that thiazole antibiotics Siomycin A and thiostrepton inhibit FoxM1 and induce apoptosis in human cancer cells. Here, we report that Siomycin A and thiostrepton stabilize the expression of a variety of proteins, such as p21, Mcl-1, p53 and hdm-2 and also act as proteasome inhibitors in vitro. More importantly, we also found that well-known proteasome inhibitors such as MG115, MG132 and bortezomib inhibit FoxM1 transcriptional activity and FoxM1 expression. In addition, overexpression of FoxM1 specifically protects against bortezomib-, but not doxorubicin-induced apoptosis. These data suggest that negative regulation of FoxM1 by proteasome inhibitors is a general feature of these drugs and it may contribute to their anticancer properties.

## Introduction

The proteasome is a protein complex that target ubiquitin-tagged proteins for degradation in an ATP-dependent manner. Recent advances in the understanding of the mechanisms of proteasome activity led to the development of proteasome inhibitors as effective drugs against human cancer. Bortezomib (Velcade) was the first proteasome inhibitor approved for the treatment of multiple myeloma with potential benefits against other types of cancer in the future. Since certain types of cancer may rely on a functional proteasome for growth, inhibition of proteasome activity would selectively kill these tumors [1]. However, the precise mechanisms of the anticancer activity of proteasome inhibitors are still not well understood. Several explanations have been presented for the antitumor properties of proteasome inhibitors, such as NF-kB inhibition, stabilization of p53, shift in the balance between pro- and antiapoptotic Bcl-2-family proteins and other (reviewed in ref. [2]). Abnormal NF-kB regulation has been shown in a variety of cancers leading to the transcriptional activation of genes responsible for cell proliferation, inhibition of apoptosis, angiogenesis and metastasis [3]. Proteasome inhibitors hinder NF-kB transcriptional activity via stabilization of IkB and sequestration of NF-kB in the cytoplasm [1]. Significance of NF-kB targeting by bortezomib was validated in multiple myeloma cells where genes that belong to NF-kB pathway were significantly overexpressed in samples associated with response to bortezomib [3], [4]. In addition, recently it has been proposed that the anticancer effects of proteasome inhibition may depend on preventing the destruction of the CDK inhibitor, p27 [5].

Forkhead box (Fox) M1, FoxM1, is a transcription factor of the Forkhead family that induces the expression of genes involved in cell cycle progression and maintenance of genomic stability [6]. It has been shown that FoxM1 is strongly upregulated in a variety of human solid tumors (reviewed in ref. [7], [8]), while its expression is suppressed in non-dividing cells [6]. The role of FoxM1 in promoting cancer was further emphasized by the poor prognosis for breast cancer patients with higher levels of FoxM1 gene expression [9]. On the other hand, suppression of FoxM1 expression delayed liver tumor growth in mice [10], [11] and inhibited the metastatic potential of human pancreatic cancer cells in vitro [12]. Since FoxM1 suppression appears to inhibit tumorigenesis, chemical compounds that target FoxM1 may act as anticancer drugs [13], [14], [15], [16], [17]. In our previous studies we demonstrated that thiazole antibiotics Siomycin A and thiostrepton induce apoptosis in human cancer cells and inhibit FoxM1 expression [13], [17], [18]. Here, we report that thiopeptides, Siomycin A and thiostrepton also act as proteasome inhibitors. Furthermore, we showed that well-known proteasome inhibitors such as MG115, MG132 or Bortezomib inhibit FoxM1 transcriptional activity and expression. We propose that negative regulation of FoxM1 by proteasome inhibitors may contribute to their anticancer properties.

## Results and Discussion

**Figure 1:**
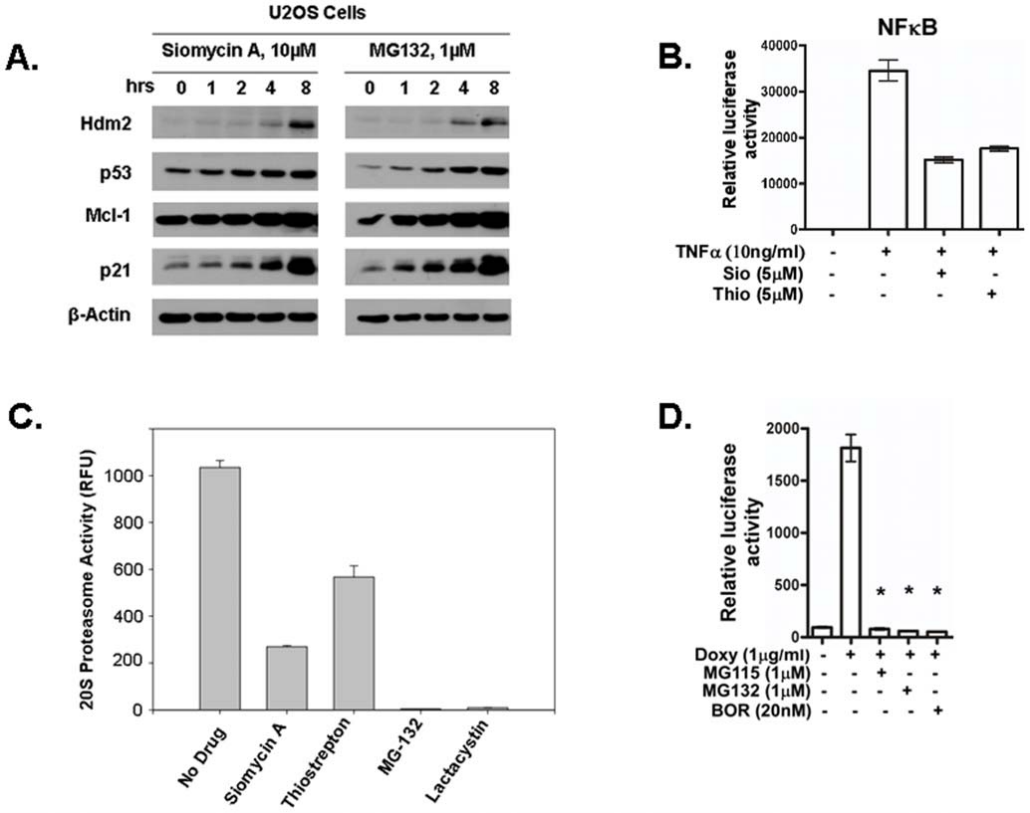
Thiazole antibiotics act as proteasome inhibitors and the known proteasome inhibitors target FoxM1. (A) Siomycin A stabilizes protein expression similarly to proteasome inhibitor MG132. U2OS cells treated as indicated were harvested and immunoblotting was carried out with antibodies specific for p53, p21, hdm2, Mcl-1, and β-actin. (B) Thiazole antibiotics inhibit NF-kB transcriptional activity. 293T-NF-κB-Luc cells were induced with 10 ng/mL TNF-α for 24 hrs and treated with 5 µM of the thiazole antibiotics for additional 10 hrs followed by luciferase assay. The results shown are mean±SD of three separate experiments. (C) Inhibition of 20S proteasome activity by Siomycin A and thiostrepton. The inhibition of proteasome activity by the thiazole antibiotics *in vitro* was less efficient compared with proteasome inhibitors, MG-132 and lactacystin as detected by using 20S Proteasome activity kit (Millipore). The free AMC fluorescence was quantified at 380/460 nM in a fluorometer (SpectraMax GeminiXS, Molecular Devices). The results shown are mean±SD of three separate experiments. (D) Proteasome inhibitors inhibit FoxM1 transcriptional activity. C3-Luc cells were treated with a combination of 1 ug/ml doxycycline and the indicated concentrations of the proteasome inhibitors MG115, MG132 and bortezomib (BOR). The luciferase activity was determined by using the Luciferase Assay System (Promega) according to the manufacturer's instructions. The results shown are mean±SD of three independent experiments. The asterisks on each graph represent statistically significant differences with *p* values<0.001.

Previously, we have shown that Siomycin A and thiostrepton inhibit FoxM1 transcriptional activity [13], [17], [18]. We also detected decrease in FoxM1 protein levels after thiopeptide treatment [13], [17], [18] as a result of FoxM1 positive autoregulatory loop [19]. However, when we evaluated the expression of other cellular proteins following exposure to thiazole antibiotics we found that Siomycin A treatment led to an opposite effect, predominantly to the stabilization of a variety of proteins, such as p21, Mcl-1, p53 and hdm2 (Fig. 1A). It has been shown earlier that these proteins are usually upregulated by proteasome inhibitors [2], [20] and we found that Siomycin A and proteasome inhibitor MG132 stabilize the expression of these proteins in a similar manner (Fig. 1A). This was the first evidence suggesting that thiazole antibiotics may also inhibit proteasome activity.

Since proteasome inhibitors usually hinder the activity of NF-κB via the stabilization of its negative regulator IkB-α [1], [2] we examined whether Siomycin A and thiostrepton inhibit NF-kB activity. 293T cells stably expressing an NF-kB-Luc reporter construct were treated with TNF-α and the next day with Siomycin A or thiostrepton, and the luciferase activity was measured. We found that both thiostrepton and Siomycin A suppress NF-kB transcriptional activity (Fig. 1B) suggesting that thiazole antibiotics inhibit NF-kB activity similarly to proteasome inhibitors [21].

To directly test whether the thiazole antibiotics inhibit proteasome activity in vitro we compared them with well-known proteasome inhibitors MG132 and lactacystin against the 20S proteasome using the Proteasome Activity Assay Kit (Millipore/Chemicon) (see materials and methods). We found that thiazole antibiotics are indeed proteasome inhibitors, although not as potent as bona-fide proteasome inhibitors, MG132 and lactacystin (Fig. 1C). Since we demonstrated before that the thiopeptides inhibit the transcriptional activity and the expression of FoxM1 [13], [18] and here we showed that they also act as proteasome inhibitors (Fig. 1), we decided to test the notion that other known proteasome inhibitors may inhibit FoxM1 as well. To evaluate how proteasome inhibitors affect FoxM1 transcriptional activity we used a derivative of U2OS osteosarcoma cell line, C3-Luc cells with a doxycycline-inducible FoxM1-GFP fusion protein and a firefly luciferase under the control of multiple FoxM1 response elements [13]. Cells were treated with a combination of doxycycline and proteasome inhibitors, and 24 hours later the luciferase activity was measured. We confirmed that all tested proteasome inhibitors strongly inhibited FoxM1 transcriptional activity (Fig. 1D) similarly to the thiazole antibiotics [13], [17], [18].

**Figure 2:**
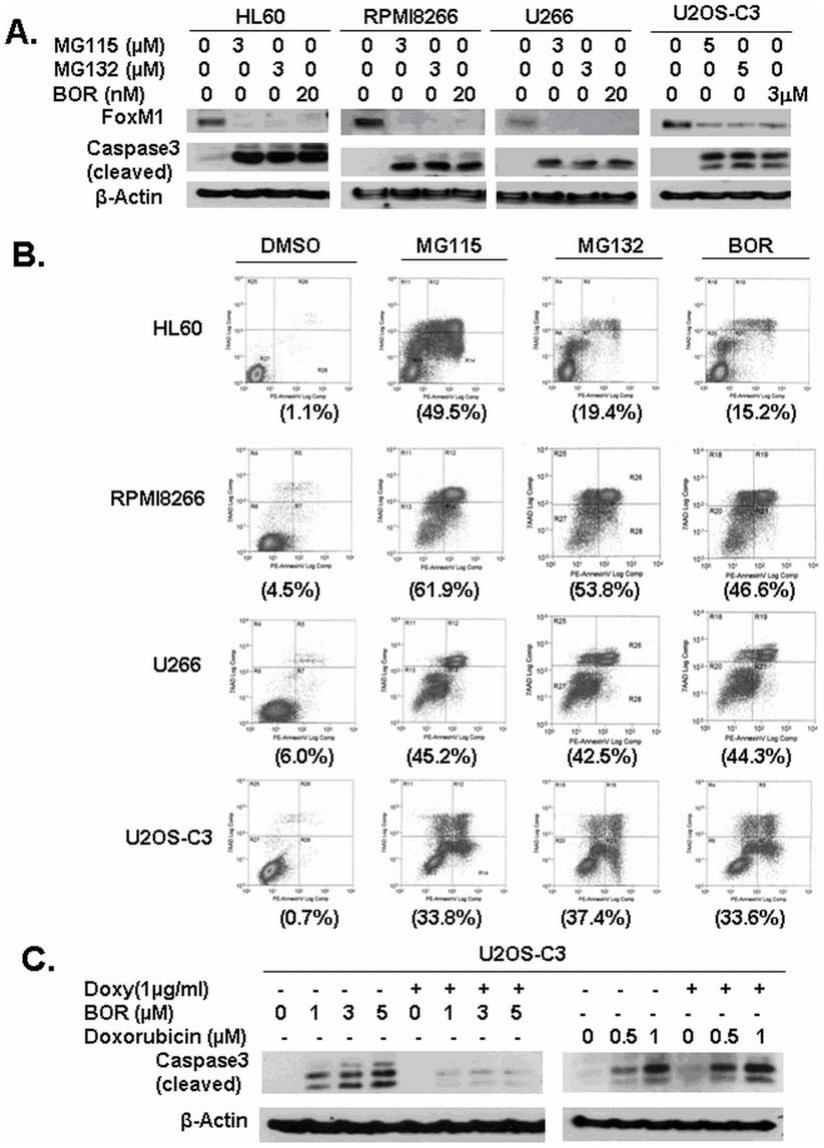
Proteasome inhibitors target FoxM1 protein and overexpression of FoxM1 partially protects cancer cell lines from proteasome inhibitor-induced apoptosis. (A) Proteasome inhibitors induce apoptosis and inhibit FoxM1 protein expression in multiple myeloma, leukemia and osteosarcoma cell lines. U266 and RPMI8226 multiple myeloma, HL-60 leukemia and U2OS-C3 osteosarcoma cell lines were treated with proteasome inhibitors MG115, MG132 and bortezomib (BOR). Apoptosis was assessed by immunoblotting for cleaved caspase-3 and FoxM1 protein levels were detected by immunoblotting. β-actin was used as the loading control. (B) Proteasome inhibitor-induced apoptosis was further quantified by using Annexin V -PE/7AAD staining. U266 and RPMI8226 multiple myeloma, HL-60 leukemia and U2OS-C3 osteosarcoma cell lines were grown in the absence or presence of indicated concentrations of proteasome inhibitors MG115, MG132 and bortezomib (BOR) for 24 hours. Following drug treatment cells were stained with Annexin V- PE/7AAD and then analyzed by flow cytometry. Percentage of apoptotic cells is shown in brackets. Concentrations of drugs are the same as in the Fig 2A. (C) Overexpression of FoxM1 specifically protects against cell death induced by Bortezomib. Overexpression of FoxM1 protected against cell death induced by increasing amount of bortezomib (BOR), but not that of doxorubicin. Immunoblotting was carried out with antibodies specific for cleaved caspase-3 and β-actin.

In addition, we investigated if proteasome inhibitors suppress FoxM1 protein expression as well. We treated U266 and RPMI8226 multiple myeloma cells, HL-60 leukemia cells and human U2OS-C3 osteosarcoma cells with proteasome inhibitors MG115, MG132 and bortezomib (Fig. 2A) and analyzed the cell lysates for the levels of FoxM1 by immunoblotting. We found that proteasome inhibitors repress FoxM1 protein expression, suggesting that they antagonize not only the transactivation ability of FoxM1, but they also inhibit its expression (Fig. 2A) very similarly to Siomycin A and thiostrepton [13], [17], [18]. Moreover, we found by immunoblotting for cleaved caspase-3 and also by annexin V staining that proteasome inhibitors induce apoptosis in a variety of human cancer cell lines (Fig. 2A, B), which correlated with the suppression of FoxM1 (Fig. 2A).

To further examine the role of FoxM1 in apoptosis induced by proteasome inhibitors we utilized a U2OS-C3-osteosarcoma cell line with a doxycycline-inducible FoxM1-GFP fusion protein [13] and tested how proteasome inhibitors induce cell death in the presence and the absence of exogenous FoxM1. FoxM1 expression was induced with the addition of doxycycline and the following day the cells were treated with different concentrations of bortezomib for 24 hours (Fig. 2C). We observed that overexpression of FoxM1 protected cells against cell death induced by bortezomib as detected by immunoblotting for cleaved caspase-3 (Fig. 2C). In contrast, we found that FoxM1 does not protect against doxorubicin-induced apoptosis (Fig. 2C), suggesting that FoxM1 specifically protects cells against proteasome inhibitor-induced apoptosis. Since proteasome inhibitors down-regulate FoxM1 and FoxM1 overexpression protects against cell death mediated by proteasome inhibitors, suppression of FoxM1 may be required for the anticancer activity of these drugs. Further experiments are needed to clarify the mechanisms of FoxM1 down-regulation by proteasome inhibitors and the importance of FoxM1 suppression in the activity of proteasome inhibitors as anticancer drugs.

## Materials and Methods

### Cell lines, media and chemical compounds

U266 and RPMI8226 multiple myeloma cell lines, and HL-60 leukemia cell line were purchased from American Type Culture Collection and were grown in RPMI1640 medium (Invitrogen). A U2OS clone C3 cell line with doxycycline-inducible FoxM1-GFP fusion protein [10]; U2OS derived C3-Luc osteosarcoma cell line stably expressing the doxycycline-inducible FoxM1-GFP and firefly luciferase under the control of multiple FoxM1 responsive elements [13] and 293T-NF-κB-Luc cell line stably expressing an NF-kB-Luc reporter were grown in DMEM medium (Invitrogen). In all cases the media were supplemented with 10% fetal bovine serum (Atlanta Biologicals) and 1% penicillin-streptomycin (GIBCO) and the cell lines were kept at 37°C in 5% CO_2_. Cell lines were tested for mycoplasma contamination using MycoAlert Mycoplasma detection kit (Lonza Rockland) and were found to be negative. Siomycin A (NCI), thiostrepton (Sigma), doxorubicin (Sigma), MG115 (Sigma), MG132 (Calbiochem) and bortezomib (Velcade) (Millenium Pharmaceuticals) were dissolved in DMSO (dimethylsulfoxide), doxycycline (Clontech) in PBS (phosphate-buffered saline) and TNF-α (R&D Systems) in PBS containing 0.1% bovine serum albumin.

### Immunoblot analysis

Cancer cells of different origin treated as indicated were harvested and lysed by using IP buffer (20 mM HEPES, 1% Triton X-100, 150 mM NaCl, 1 mM EDTA, 1 mM EGTA, 100 mM NaF, 10 mM Na_4_P_2_O_7_, 1 mM sodium othrovanadate, 0.2 mM PMSF supplemented with protease inhibitor tablet (Roche Applied Sciences). Protein concentration was determined by the Bio-Rad Protein Assay (BIO-RAD). Isolated proteins were separated on 8% or 10% SDS-PAGE and transferred to PVDF membrane (Millipore). Immunoblotting was carried out as described in [22], [23], [24] with antibodies specific for FoxM1 (a gift from Dr. Costa's lab), cleaved caspase-3 (Cell signaling), p53 (Santa-Cruz Biotechnology), p21 (BD-Pharmingen), hdm2 (Santa-Cruz Biotechnology), Mcl-1 (LabVision), and β-actin (Sigma).

### Luciferase assays

The derivative of U2OS osteosarcoma cell line (U2OS-C3-Luc) was treated with a combination of 1 µg/ml doxycycline and the indicated concentrations of the proteasome inhibitors MG115, MG132 and bortezomib. Also, the 293T-NF-κB-Luc cell line was stimulated with 10 ng/mL TNF-α for 24 hrs followed with 5 µM of Siomycin A or thiostrepton treatment for 10 hrs, respectively. The luciferase activity was determined by using the Luciferase Assay System (Promega) according to the manufacturer's instructions and data were normalized on the amount of protein in the samples.

### 20S proteasome activity assay

The inhibition of proteasome activity by thiazole antibiotics *in vitro* was compared with that of two known proteasome inhibitors, MG-132 and lactacystin using 20S Proteasome Activity Assay Kit (Millipore) according to the instructions of the manufacturer. The assay is based on the detection of the fluorophore 7-amino-4-methylcoumarin (AMC) after cleavage from the labeled substrate LLVY-AMC in the presence and the absence of tested compounds. 25 µM of the compounds were pre-incubated with the proteasome samples for 15 minutes at room temperature and then the proteasome substrate was added. Samples were incubated for 1 hour at 37°C and the free AMC fluorescence was quantified at 380/460 nm in a fluorometer (Spectra Max GeminiXS, Molecular Devices).

### Annexin V-PE/7AAD staining

Control and treated cells were stained using Annexin V-PE apoptosis detection kit (BD-Pharmingen) according to the manufacturer's recommendations as described in [17].

### Statistical analysis

Statistical analysis was performed with Microsoft Excel using the Student *t* test. *P* values of<0.05 were considered to be statistically significant.
